# A Safety Evaluation of the Impact of Maternity-Orientated Human Factors Training on Safety Culture in a Tertiary Maternity Unit

**DOI:** 10.1097/PTS.0000000000000609

**Published:** 2019-05-30

**Authors:** Sophia P. Ansari, Malissa E. Rayfield, Victoria A. Wallis, Jennifer E. Jardine, Edward P. Morris, Edward Prosser-Snelling

**Affiliations:** From the ∗ Department of Obstetrics and Gynaecology, Norfolk & Norwich University Hospital; † Norfolk & Norwich University Hospital, Norwich; ‡ Clinical Fellow at the National Maternity and Perinatal Audit Royal College of Obstetricians and Gynaecologists (RCOG), London; § Each Baby Counts, Royal College of Obstetricians and Gynaecologists (RCOG), London, United Kingdom.

**Keywords:** human factors, patient safety, training, maternity

## Abstract

Human factors have risen to attention in maternity as key contributors to patient harm. Despite national recommendation for multidisciplinary human factors training, there is a lack of guidance and healthcare-orientated training.

**Objectives:**

The aim of the study was to evaluate the impact of maternity-orientated human factors training program on safety culture in a tertiary maternity unit.

**Methods:**

This prospective observational cohort study was conducted for 6 months in a tertiary maternity unit. Participants involved in high-risk intrapartum care completed the Hospital Survey of Patient Safety Culture before and after intervention. Statistical analysis was performed using the χ^2^ test with statistical significance at 5% (*P* = 0.05).

**Intervention:**

The human factors curriculum included situational awareness, communication, decision-making, conflict resolution, teamwork, and leadership. A train-the-trainer approach generated a faculty to disseminate multidisciplinary training. Traditional classroom teaching, social media content, and cognitive activities provided theoretical foundations. Forum theater and behavioral simulation taught complex communication issues. Regular labor ward simulations helped embed training into clinical practice.

**Results:**

The results demonstrated statistically significant improvement in safety culture domains of communication openness, handover, nonpunitive response to error, and overall safety perception. Participants felt more able to challenge decisions or actions of those in authority, 33% responded “most of the time or always” in August increasing to 42% in January with a reduction of 50% in those responding “never” (*P* = 0.02). No change was found relating to team working, staffing or manager expectations promoting patient safety.

**Conclusions:**

This study is proof-of-concept that maternity-orientated human factors training can improve safety culture.

## Abstract 2

Supplemental digital content is available in the text.

Human factors have long been recognized by other safety critical industries as key contributing factors in adverse events.^1^ These include breakdown in communications, poor decision-making, lack of clear leadership, and team working. In medicine, human factors, also referred to as nontechnical skills, are widely acknowledged as contributing to medical harm.^2^

These factors have risen to national attention in maternity care through high-profile cases, such as the Kirkup report, and confidential enquires by the Royal College of Obstetricians and Gynaecologists, such as Each Baby Counts and Mothers and Babies Reducing Risk through Audits and Confidential Enquiries across the United Kingdom.^3–5^ These have provided invaluable information on the cause of intrapartum and neonatal death, with data analysis focusing on individual and organizational factors, instead of simply clinical events.

Despite the national recommendation for multidisciplinary training, there is no formal midwifery or medical curriculum or training and consequently a lack of standardization between units and specialities.^6^ Formal training, equivalent to crew resource management used in aviation, has been proposed as the solution for healthcare.^7^ Safety critical industries, such as nuclear and aviation, often experience similar organizational and therefore cultural issues, which allows for direct transfer of some training across all work settings.^8,9^ Although this addresses the basic principles of human factors, such as team-working, leadership, communication, decision-making, and situational awareness, it is essential to acknowledge that flying a plane and delivering babies are different.^10^ In the National Health Service (NHS), and specifically maternity, there is more diversity in work settings (with midwifery standalone units and tertiary teaching hospitals) culture and even organizational aspirations. This means that the “one-size-fits-all” training model is ineffective and problematic. There is a growing consensus that human factors training should be specific and relatable to healthcare to elicit the benefits achieved in aviation.^11^

Adverse events in maternity are rare but often complex and multifactorial. Therefore, it is difficult to attribute causality of a potential reduction in these events simply because of human factors training.

Safety culture is defined as “the product of individual and group values, attitudes, perceptions, competencies, and patterns of behavior that determine the commitment to, and the style and proficiency of, an organization's health and safety management.”^12^ It measures team-working, leadership, handover, and communication, which are all key elements in human factors. Positive patient safety culture is associated with fewer adverse events, which can be used as a surrogate marker of patient safety.^13^

The overall aim of this study was to determine whether the implementation of a maternity-orientated human factors training program could improve safety culture. The objectives were (*a*) to establish the safety culture in the maternity unit, (*b*) design and implement a sustainable maternity-orientated human factors training program, and (*c*) generate a faculty of trainers from multidisciplinary healthcare professionals to deliver the training.

## METHODS

### Study Design

This was a prospective observational cohort study conducted for a 6-month period from August 1, 2017 to January 31, 2018. The study period commenced after maternity staff had rotated into new areas to reduce potential loss to follow-up but acknowledge that some staff were recruited to the trust after this time. A confounding factor is that some maternity staff had already received training in human factors. This was limited to three consultant obstetricians and three consultant anesthetists.

### Population

The study was conducted at the Norfolk and Norwich University Hospital NHS Trust, a tertiary maternity and neonatal unit with an average birth rate of 5600 deliveries per year. All healthcare professionals who regularly worked on delivery suite involved in high-risk intrapartum care^14^ during the study period were eligible to participate, including obstetricians, midwives, healthcare assistants, anesthetists, theater practitioners, and neonatologists. Staff working outside intrapartum care were excluded from the study to maintain a specific cohort to establish association. Nonpermanent staff were also excluded because of lack of exposure to unit culture.

A cohort of participants received basic human factors training and the opportunity to become trainers. This was advertised through posters, e-mails, social media pages, and announcements during shift handover. All staff eligible to participate including key senior staff, such as consultant obstetricians, anesthetists, and coordinating midwives were invited to self-nominate. This resulted in a cohort of 61 participants, a mixed cross-section of the population covering all range of shift patterns. Table 1 represents the total number of staff with corresponding professional role. The cohort of participants who received human factors training were a mixture of midwives, theater staff, midwifery care assistants, neonatologists, anesthetists, and obstetricians—who composed of both consultant obstetricians and speciality trainees in obstetrics and gynecology.

**TABLE 1:** Cohort of Participants Who Received Human Factors Training to Become the Body of Educators Divided Into Professional Group

Healthcare Professional	Cohort	Population
Midwife	30	152
Obstetrician	19	38
Theatre staff	3	20
Midwifery care assistant	2	17
Neonatologist	3	27
Anesthetist	4	15
Total	61	296

### Educational Intervention

The cohort received human factors training in theoretical concepts over a 2-day course delivered by an external consultancy firm, Atrainability. The company specializes in human factors training as the cofounder contributed to the introduction of the subject into British Airways as a method of reducing “pilot error.” All facilitators are experienced in human factors from civil and military aviation backgrounds. The authors preassessed Atrainability's materials to ensure that they were in reflection of themes highlighted as contributory factors in intrapartum stillbirth, early neonatal death, and hypoxic brain injury—shown in reports such as the Royal College of Obstetricians and Gynaecologists Each Baby Counts Report 2015. The course curriculum covered the following topics: safety culture, migration of boundaries, cognition, human limitations, stress & team dynamics, situational awareness, self-awareness, decision-making, dealing with difficult behavior, communication, leadership, handover/safety huddles, and debriefing/feedback. Educational methods included interactive lectures and small group exercises. Participants were then encouraged to self-nominate to create a faculty of 17 multidisciplinary trainers from this initial cohort of 61 to deliver the training to the remaining population.

### Theoretical Training

The training was disseminated to the population using a blended learning approach. Basic concepts of human factors were introduced through classroom teaching in monthly mandatory drills and skills sessions. This consisted of short cognitive activities to illustrate a human factor element, with maternity examples to demonstrate relevance in clinical practice. Social media–delivered content, for example “The Little Voice Inside” video from the University Hospital of Leicester, promoted identification, reflection, and group discussion of concepts, such as situational awareness, task fixation, confirmation bias, and mental models. Forum theater^15^ and behavioral simulations demonstrated by the faculty explored more complex communication issues, such as conflict resolution and transactional analysis, and provided an opportunity for participants to generate solutions in a psychologically safe space. The training specifically focused on empowering staff to raise concerns through structure communication tools for graded assertiveness, for example, PACE or Probe, Alert, Challenge, and Emergency. This was complimented with the briefing of senior staff to acknowledge and appropriately manage the safety concern, rather than dismiss the challenge.

### Simulation Training

The focus of training was primarily through impromptu in situ simulations on the delivery suite. This ranged from large-scale obstetric emergencies to small one-on-one “micro” simulations, for example, distractions and interruptions while taking a blood sample. These micro simulations clearly demonstrate the human factors relevant in clinical practice and more importantly provide opportunity for participants to practice behaviors and management of these situations to reduce future error. The maternity unit did not have a designated area for training. The simulations were specifically designed to only last 15 to 20 minutes, including the set-up and debriefing to prevent disruption to patient care and the maternity service. The simulations were delivered by faculty members and occurred at least once a week during the day, night, and weekend shift patterns to include all staff groups. The project devised strict criteria for when an impromptu simulation would not be appropriate (Appendix 1, http://links.lww.com/JPS/A233).

### Control

The study did not have a control group because of resource limitation and service demand. Specifically, the lack of maternity staff would reduce the cohort and influence the power of the results.

### Outcome

The primary outcome measure was the Hospital Survey of Patient Safety Culture Agency for Healthcare Research and Quality,^16^ a psychometrically validated tool to assess safety culture specifically in acute care. The survey contains 42 items measured on five-point Likert response scale of agreement (strongly agree to strongly disagree) and frequency (never to always). These items were categorized into the 12 domains, which constitute safety culture. Compared with other safety assessment tools, the survey has multiple subscales measuring each dimension to increase reliability of results.^17^

Secondary outcome measures included frequency of reporting adverse outcomes in maternity as suggested by Maternity Review.^18^ This is difficult to interpret as improvement in patient safety can be correlated to an increase in reporting, rather than a reduction. Error reporting is a key component of any organization to understand the patient safety issues. As organizations address and improve safety culture, the type of events reported change from large events that result in significant harm to multiple, “minor” errors. Higher reporting rates are associated with more positive safety culture.^19^

Training quality was assessed using the Kirkpatrick model of training evaluation.^20^ The model is widely used for its simplistic and pragmatic approach and allows for evaluation of different training styles.^21^

### Ethical Consideration

Ethical approval was not deemed necessary because this was an evaluation of the efficacy of this particular quality improvement program before wider rollout.

### Survey Administration

The survey was conducted at three set points at 2 monthly intervals representing the beginning, mid, and end points of the project. Each data set spanned a 3-day period to include day, night, and weekend staff perceptions. This consisted of a total of 18 handovers from which data were collected from participants. The survey was administered through the use of portable electronic tablets to improve participant response rate. Individual identifiers were not required in the survey because there was a single individual for data collection to prevent duplication and to allow for anonymity and thus reduced bias. The survey asked participants to define their healthcare role for each data set. There were two participants in the January data set who did not answer this question, represented as “skipped” in Table 2.

**TABLE 2:** Response to Question 1: ‘Which best describes your role?’

Healthcare Professional	August	November	January	Population
Midwife	36	49	46	152
Obstetrician	15	26	16	38
Theater staff	8	12	7	20
Midwifery care assistant	6	9	8	17
Neonatologist	2	1	2	27
Anesthetist	3	6	4	15
Answered	70	103	83	296
Skipped	0	0	2	

### Statistical Analysis

The answer response was aggregated into broader categories of “disagree,” “neither” and “agree,” or “never”/“rarely,” “sometimes,” and “often”/“always.” This was required for the statistical analysis because often there was no response in the stronger preference categories, such as strongly agree or strongly disagree. Preintervention and postintervention group comparison was conducted using χ^2^ test for nonordered category variables with statistical significance at the 5% level (*P* < 0.05). A pilot study in May 2017 demonstrated poor participant engagement because of concerns about anonymity of responses. The electronic survey collection tool resolved this issue; however, this meant that the Cronbach α to assess internal reliability was not applicable in this context.

## RESULTS

### AHRQ: Hospital Survey of Patient Safety Culture

#### Participant Characteristics

The demographic characteristics are presented in Table 2. There were more obstetricians in the second and third data set due to multiple obstetrician handovers and increased attendance at daily cesarean section operating lists because of complex cases. Neonatology involvement was lower than expected because of time limitations on the delivery suite and workload. Statistical analysis performed using χ^2^ test for comparison of group composition demonstrated no significant difference between the groups (*P* = 0.09).

### Unit-Level Aspects of Patient Safety Culture

The unit-level domains assess the safety culture in relation to the labor ward. The participants responded positively to the domain “teamwork” representing cooperation and respect, which was unchanged after the intervention. For “supervisor/manager expectations and actions promoting patient safety,” participant response was marginally positive but did not increase with the intervention. There was an overall improvement in “organizational learning” with 83% of participants in agreement with the statement “we are actively doing things to improve patient safety” compared with 78% preintervention. However, this was not deemed statistically significant. The “Overall perception of safety” was positive, with statistical significance at the 5% level. This was evident in the response to the statement “we have a patient safety problem in this unit” with 45% of participants actively disagreeing compared with 30% (*P* < 0.01). There was evidence of a trend that participants felt more informed of errors and were invited for discussion about prevention postintervention in the domain “feedback and communication about error.” However, this finding was not statistically significant. In the domain “communication openness,” there was evidence of a trend that participants felt more positive and willing to raise concerns and question the actions of those in authority, which might affect patients. The responses to these statements were statistically significant. Staffing levels were a key issue from the response of the participants, which remained unchanged during the study period. There was a positive response to the domain “nonpunitive response to error.” Fewer participants were in agreement with the statement “when an event is reported, it feels like the person is being written up, not the problem” 32% postintervention compared with 42%, statistically significant at the 5% level. Participants were required to grade the unit on patient safety, with an increase in “excellent” or “good” response from 45% to 73% postintervention, a finding that was statistically significant.

### Hospital-Level Aspects of Patient Safety Culture

There are three domains to assess safety culture at hospital level. Overall, there was an increase in positive response in all domains after the training intervention. The domain “hospital management support for patient safety” indicates managerial influence on work climate. In particular, fewer participants actively disagree with the statement *“hospital management seems interested in patient safety only after an adverse event”* 56% decreased to 49%, which was statistically significant at the 5% level (*P* < 0.05). With regard to the domain “teamwork across units,” there was an increase in positive response, especially in the statement *“hospital units work well together to provide the best care for patients.”* After intervention, 68% of participants agree with the statement compared with 53% (*P* < 0.05).

Medical errors and adverse events have been attributed to poor communication at handover and shift change.^22^ There was an increase in positive response in all statements regarding handover correlating with statistical significance at the 5% level (*P* < 0.05).

### Frequency of Event Reporting

Fifty percent of participants responded “most of the time” or “always” to reporting a mistake, regardless of the consequences to patient safety. This remained unchanged during the study period.

There was evidence of an increase of participants who had not reported a single event for the last 12 months; however, there was also an increase in those reporting at higher rates (11–20 reports) (Table 3).

**TABLE 3:** Results From the Hospital Survey of Patient Safety Culture

Question	Answer Choice	August	November	January	*P* (<0.05)
*People support one another in this unit*	Agree	84% (57)	93% (79)	86% (65)	0.22
	Neither	9% (6)	1% (1)	9% (7)	
	Disagree	7% (5)	6% (5)	5% (4)	
*We have enough staff to handle the workload*	Agree	22% (15)	14% (12)	16% (12)	<0.01
	Neither	2% (1)	7% (6)	6% (5)	
	Disagree	76% (51)	79% (68)	78% (59)	
*When a lot of work needs to be done quickly, we work together as a team to get the work done.*	Agree	87% (59)	91% (77)	90% (68)	0.75
	Neither	6% (4)	6% (5)	5% (4)	
	Disagree	7% (5)	3% (3)	5% (4)	
*In this unit, people treat each other with respect.*	Agree	78% (52)	81% (70)	76% (57)	0.94
	Neither	13% (9)	14% (12)	15% (11)	
	Disagree	9% (6)	5% (4)	9% (7)	
*Staff in this unit work longer hours than is best for patient care*	Agree	64% (43)	61% (52)	65% (49)	0.97
	Neither	16% (11)	27% (23)	17% (13)	
	Disagree	20% (13)	12% (10)	18% (14)	
*We are actively doing things to improve patient safety*	Agree	78% (53)	87% (75)	83% (63)	0.19
	Neither	12% (8)	12% (10)	13% (10)	
	Disagree	10% (7)	1% (1)	4% (3)	
*We use more agency/temporary staff than is best for patient care*	Agree	22% (15)	12% (10)	10% (8)	0.29
	Neither	8% (5)	20% (17)	20% (15)	
	Disagree	70% (47)	68% (59)	70% (53)	
*Staff feel like their mistakes are held against them*	Agree	36% (24)	34% (29)	32% (24)	0.64
	Neither	22% (15)	30% (26)	26% (20)	
	Disagree	42% (28)	36% (31)	42% (32)	
*Mistakes have led to positive changes here*	Agree	63% (43)	77% (66)	75% (56)	0.12
	Neither	28% (19)	19% (16)	20% (15)	
	Disagree	9% (6)	4% (4)	5% (4)	
*It is just by chance that more serious mistakes don't happen around here*	Agree	42% (28)	46% (39)	30% (23)	<0.01
	Neither	24% (16)	16% (14)	19% (14)	
	Disagree	34% (23)	38% (32)	51% (39)	
*When one area in this unit gets really busy, others help out*	Agree	49% (33)	61% (53)	52% (39)	0.79
	Neither	15% (10)	20% (17)	16% (12)	
	Disagree	36% (24)	19% (16)	32% (24)	
*When an event is reported, it feels like the person is being written up, not the problem*	Agree	42% (28)	34% (29)	32% (24)	0.03
	Neither	18% (12)	28% (24)	29% (22)	
	Disagree	40% (27)	38% (33)	39% (29)	
*After we make changes to improve patient safety, we evaluate their effectiveness*	Agree	54% (37)	69% (59)	66% (50)	0.13
	Neither	27% (18)	15% (13)	21% (16)	
	Disagree	19% (13)	16% (14)	13% (10)	
*We work in crisis mode trying to do too much, too quickly*	Agree	55% (37)	62% (53)	61% (46)	0.50
	Neither	15% (10)	21% (18)	11% (8)	
	Disagree	30% (20)	17% (14)	28% (22)	
*Patient safety is never sacrificed to get more work done*	Agree	34% (13)	44% (38)	38% (29)	0.37
	Neither	16% (11)	16% (14)	20% (15)	
	Disagree	50% (34)	40% (34)	42% (32)	
*Staff worry that mistakes they make are kept in their personal files*	Agree	51% (34)	40% (34)	46% (35)	0.13
	Neither	28% (19)	34% (29)	24% (18)	
	Disagree	21% (14)	26% (23)	30% (23)	
*We have patient safety problems in this unit*	Agree	48% (32)	33% (28)	31% (24)	<0.01
	Neither	22% (15)	22% (19)	24% (18)	
	Disagree	30% (20)	45% (39)	45% (34)	
*Our procedures and systems are good at preventing errors from happening*	Agree	54% (37)	71% (61)	67% (51)	0.08
	Neither	29% (20)	21% (18)	22% (17)	
	Disagree	17% (11)	8% (7)	11% (8)	
*My supervisor/manager says a good work when he/she sees a job done according to established patient safety procedures.*	Agree	59% (39)	58% (51)	56% (42)	0.87
	Neither	16% (11)	16% (14)	16% (12)	
	Disagree	25% (17)	26% (23)	28% (21)	
*My supervisor/manager seriously considers staff suggestions for improving patient safety*	Agree	52% (35)	62% (54)	60% (45)	0.40
	Neither	26% (17)	16% (14)	21% (16)	
	Disagree	22% (15)	22% (19)	19% (14)	
*Whenever pressure build up, my supervisor/manager wants us to work faster, even if it means taking shortcuts*	Agree	36% (24)	33% (29)	32% (24)	0.78
	Neither	13% (9)	23% (20)	15% (11)	
	Disagree	51% (34)	44% (38)	53% (40)	
*My supervisor/manager overlooks patient safety problems that happen over and over*	Agree	21% (14)	15% (13)	16% (12)	0.53
	Neither	20% (13)	15% (13)	20% (15)	
	Disagree	59% (39)	70% (60)	64% (48)	
*We are given feedback about changes put into place based on event reports*	Never/rarely	15% (10)	14% (12)	5% (4)	0.06
	Sometimes	37% (25)	29% (25)	45% (33)	
	Most times/always	48% (20)	57% (48)	50% (37)	
*Staff will freely speak up if they see something that may negatively affect patient care*	Never/rarely	3% (2)	6% (5)	7% (5)	0.06
	Sometimes	42% (28)	32% (28)	32% (24)	
	Most times/always	55% (37)	62% (53)	61% (45)	
*We are informed about errors that happen in this unit*	Never/rarely	9% (6)	8% (7)	7% (5)	0.80
	Sometimes	27% (18)	19% (16)	28% (21)	
	Most times/always	64% (43)	73% (63)	65% (48)	
*Staff feel free to question the decision or actions of those with more authority*	Never/rarely	30% (20)	21% (18)	15% (11)	0.02
	Sometimes	37% (25)	41% (35)	43% (32)	
	Most times/always	33% (22)	38% (32)	42% (31)	
*In this unit, we discuss ways to prevent errors from happening again*	Never/rarely	6% (4)	6% (5)	5% (4)	0.65
	Sometimes	36% (24)	23% (20)	31% (23)	
	Most times/always	58% (39)	71% (61)	64% (47)	
*Staff are afraid to ask questions when something does not seem right*	Never/rarely	45% (30)	51% (44)	54% (40)	0.27
	Sometimes	43% (29)	37% (32)	35% (26)	
	Most times/always	12% (8)	12% (10)	11% (8)	
*When a mistake is made, but is caught and corrected before affect the patient, how often is this reported*	Never/rarely	12% (8)	22% (19)	15% (11)	0.65
	Sometimes	41% (27)	29% (25)	36% (27)	
	Most times/always	47% (31)	49% (42)	49% (36)	
*When a mistake is made, but has no potential to harm the patient, how often is the reported*	Never/rarely	21% (14)	24% (21)	23% (17)	0.78
	Sometimes	29% (19)	29% (25)	31% (23)	
	Most times/always	50% (33)	47% (40)	46% (34)	
*When a mistake is made that could harm the patient, but does not, how often is the reported*	Never/rarely	9% (6)	16% (14)	8% (6)	0.36
	Sometimes	23% (15)	26% (22)	30% (22)	
	Disagree	68% (45)	58% (49)	62% (46)	
*Please give delivery suite an overall grade on patient safety*	Excellent/good	45% (30)	58% (50)	73% (55)	0.02
	Acceptable	51% (34)	40% (35)	24% (18)	
	Poor/failing	4% (3)	2% (2)	3% (2)	
*Hospital units do not coordinate well with each other*	Agree	35% (23)	33% (28)	24% (18)	0.14
	Neither	36% (24)	26% (22)	45% (33)	
	Disagree	29% (19)	41% (34)	31% (23)	
*Things “fall between the cracks” when transferring patients from one unit to another*	Agree	50% (33)	46% (39)	30% (22)	<0.01
	Neither	14% (9)	23% (19)	36% (27)	
	Disagree	36% (24)	31% (26)	34% (25)	
*There is good cooperation among hospital units that need to work together*	Agree	41% (27)	60% (50)	50% (37)	0.09
	Neither	42% (28)	27% (23)	42% (31)	
	Disagree	17% (11)	13% (11)	8% (6)	
*Important patient care information is often lost during shift changes*	Agree	36% (24)	24% (20)	22% (16)	0.03
	Neither	20% (13)	22% (18)	24% (18)	
	Disagree	44% (29)	54% (45)	54% (40)	
*It is often unpleasant to work with staff from` other hospital units*	Agree	6% (4)	4% (3)	1% (1)	0.22
	Neither	30% (20)	20% (17)	34% (25)	
	Disagree	64% (42)	76% (64)	65% (48)	
*Problems often occur in the exchange of information across hospital units*	Agree	38% (25)	35% (29)	23% (17)	0.02
	Neither	34% (22)	33% (28)	43% (32)	
	Disagree	28% (18)	32% (27)	34% (25)	
*The actions of hospital management show that patient safety is a top priority*	Agree	47% (31)	54% (45)	54% (40)	0.10
	Neither	26% (17)	25% (21)	30% (22)	
	Disagree	27% (18)	21% (18)	16% (12)	
*Hospital management seems invested in patient safety only after an adverse event happens*	Agree	56% (44)	47% (39)	49% (36)	0.03
	Neither	8% (5)	20% (17)	22% (16)	
	Disagree	26% (17)	33% (27)	29% (22)	
*Hospital units work well together to provide the best care for patients*	Agree	53% (35)	67% (56)	68% (50)	<0.01
	Neither	38% (25)	30% (25)	32% (24)	
	Disagree	9% (6)	3% (3)	0% (0)	
*Shift changes are problematic for patients in this hospital*	Agree	32% (21)	53% (44)	26% (19)	0.03
	Neither	29% (19)	18% (15)	20% (15)	
	Disagree	39% (26)	29% (24)	54% (40)	

### Kirkpatrick Training Evaluation

### Level 1 – Reaction

The participants who attended the initial foundation course completed a questionnaire, using a five-point Likert-type scale to assess individual reactions and thoughts to the training. The response rate was 93% (57 participants). Seventy-two percent of participants thought that the course was “very enjoyable,” 25% “enjoyable,” and only 3% “enjoyed slightly.” There were no responses for “neutral” or “did not enjoy.” Eighty-one percent of the participants felt that the course was very relevant to the work environment, and 19% relevant. One-hundred percent of participants would recommend this course to a colleague.

### Level 2 – Learning

The questionnaire encouraged the cohort to provide key learning points from the training session. Qualitative analysis generated common themes. The first was communication, specifically the use of structured tools, such as SBAR (Situation, Background, Assessment and Recommendation), and the impact or influence of different forms of communication with regard to leading questions. Other themes identified include the concept of “Just Culture,” situational awareness and briefings, and finally the effect of stress and fatigue on performance.

### Level 3 – Behavior

The Hospital Survey of Patient Safety Culture assessed if training had influenced the behavior of participants and the impact on unit culture. Participants were more likely to raise patient safety concerns and question those in authority. Participants perceived less individual blame from reporting incidents; however, this did not correlate to an increase in events reported.

### Level 4 – Results

The survey demonstrated an overall improvement in safety culture in the unit. The impact of the training on frequency of adverse outcomes will require further evaluation and research over a prolonged duration.

## DISCUSSION

This study demonstrates an overall improvement in safety culture after the maternity orientated human factors training intervention, specifically with regard to communication and openness. Participants were more likely to raise patient safety concerns and question those in authority to increase the situational awareness and reduce harm. The training included education about the importance of handover as a concept, rather than “another tick-box exercise” and tools to facilitate this, which improved participant's response to patient safety and handover, improving patient care.

Shift patterns and staffing levels did not change during the study, and therefore as expected, there was no change in response to this section of the survey.

There was good team work in the unit and the training emphasized the importance of this, which remained positive throughout the study. Teamwork itself was not formally assessed based on patient outcome but rather how human factors training impacted safety culture within the maternity unit.

As previously discussed, the number of events reported by participants is a difficult measure of safety. Over the study period, there seemed to be an increased percentage of participants not reporting a single event for a 12-month period; however, an increase in those reporting at higher rates (11–20 reports). This supports the rationale that adverse outcomes in isolation are not an appropriate measure of safety culture. It does signify the NHS is still lagging behind other safety critical industries and more work is required for improvement.

### Strengths

The study was conducted in a large tertiary teaching hospital with multiple team members together with a wide variety of maternal and neonatal complex cases. There was a robust evaluation of the impact of safety culture and the quality of training delivered to inform future programs. The study demonstrates proof of concept for a specific human factors training program and therefore would benefit from further evaluation through cluster interventions in other maternity trusts.

### Limitations

The study is observational and confounding factors cannot be completely controlled for. The study team made a pragmatic assessment of the training activity and cultural influences happening at the same time as our program and did not find any which were considered likely to have an effect on culture.

A strong confounding factor is the introduction of strong local leadership in human factors, as well as the educational intervention per se. This, however, should be considered as a factor when attempting to reproduce these results.

The study incorporated perceptions from all maternity staff groups during different shift patterns; however, only a proportion of staff received the training and the overall cohort size was small and susceptible to bias because of loss to follow-up. The demographic characteristics of the population also lack ethnic diversity because only 8% are nonwhite.

The Hospital Survey of Patient Safety Culture was developed in the United States of America with a notably different healthcare system, and there is some speculation on the relevance to the NHS. Another criticism of the survey is the length and time it takes for completion with the potential effect on validity and reliability of participant response. The survey has been psychometrically analyzed by a Scottish NHS data set, which replicated the 12 original domains of the survey. It concluded that no modifications are required and use of the survey would allow for cross-national comparisons.^23^

The anonymity of data was prioritized to encourage participation and honest opinions to generate an accurate representation of safety culture. However, this meant the authors could not calculate the Cronbach α to assess the internal reliability.

### Conclusions and Recommendations

The study suggests that multidisciplinary human factors training in maternity can positively influence patient safety culture to improve patient safety and consequently reduce adverse outcomes.

Future studies of cluster implementation of the training program are required to establish if this improvement in safety culture can be replicated in other maternity unit and specialities in the NHS.

## References

[1] Reason J Human error: models and management. *BMJ*. 2000;320:768. 10720363 10.1136/bmj.320.7237.768 PMC1117770

[2] Harmer M Independent review on the care given to Mrs Elaine Bromiley on 29 March 2005. Available at: http://www.chfg.org/resources/07_qrt04/Anonymous_Report_Verdict_and_Corrected_Timeline_Oct_07.pdf. Accessed June 2018.

[3] Kirkup B *The Report of the Morecambe Bay Investigation March 2015*. UK: The Stationery Office; 2015 ISBN 9780108561306.

[4] Royal College of Obstetricians and Gynaecologists *Each Baby Counts: 2015 Full Report*. London: Royal College of Obstetricians and Gynaecologists; 2017.

[5] Draper ES Kurinczuk JJ Kenyon S eds. on behalf of MBRRACE-UK *MBRRACE-UK Perinatal Confidential Enquiry: Term, Singleton, Normally Formed, Antepartum Stillbirth*. Leicester: The Infant Mortality and Morbidity Studies, Department of Health Sciences, University of Leicester; 2015.

[6] Greig PR Higham H Vaux E Lack of standardisation between specialties for human factors content in postgraduate training: an analysis of specialty curricula in the UK. *BMJ Qual Saf*. 2015;24:558–560. 10.1136/bmjqs-2014-003684 26041812

[7] Kohn LT Corrigan JM, Donaldson MS (Institute of Medicine) *To Err Is Human: Building a Safer Health System*. Washington, DC: National Academy Press; 2000. 25077248

[8] McCulloch P Mishra A Handa A, The effects of aviation-style nontechnical skills training on technical performance and outcome in the operating theatre. *Quality Saf Health Care*. 2009;18:109–115. 10.1136/qshc.2008.032045 19342524

[9] Catchpole KR Dale TJ Hirst DG, A multicenter trial of aviation-style training for surgical teams. *J Patient Saf*. 2010;6:180–186. 20802280 10.1097/PTS.0b013e3181f100ea

[10] Kapur N Parand A Soukup T, Aviation and healthcare: a comparative review with implications for patient safety. *JRSM Open*. 2016;7:2054270415616548. 26770817 10.1177/2054270415616548 PMC4710114

[11] de Korne DF van Wijngaarden JD Hiddema UF, Diffusing aviation innovations in a hospital in the Netherlands. *Jt Comm J Qual Patient Saf*. 2010;36:339–347. 20860239 10.1016/s1553-7250(10)36051-x

[12] Health and Safety Commission *Third Report: Organising for Safety, ACSNI Study Group on Human Factors*. London: HMSO; 1993.

[13] Mardon RE Khanna K Sorra J, Exploring relationships between hospital patient safety culture and adverse events. *J Patient Saf*. 2010;6:226–232. 21099551 10.1097/PTS.0b013e3181fd1a00

[14] National Institution for Clinical Excellence (NICE) *Intrapartum Care for Healthy Women and Babies*. London: NICE; 2014.

[15] Augusto *Theatre of the Oppressed*. New York, United States: Theatre Communications Group; 2013.

[16] Agency for Healthcare Research and Quality, Hospital Survey of Patient Safety Culture. 2016 Available at: http://www.ahrq.gov/professionals/quality-patient-safety/patientsafetyculture/hospital/index.html. Accessed June 5, 2018.

[17] Hodgen A Ellis L Churruca K, *Safety Culture Assessment in Health Care: A Review of the Literature on Safety Culture Assessment Modes*. Sydney: Australian Commission on Safety and Quality in Healthcare; 2017.

[18] National Maternity Review *Better Births – Improving outcomes of maternity services in England*. National Health Service; 2016.

[19] Hutchinson A Young TA Cooper KL, Trends in healthcare incident reporting and relationship to safety and quality data in acute hospitals: results from the National Reporting and Learning System. *Qual Saf Health Care*. 2009;18:5–10. 19204125 10.1136/qshc.2007.022400

[20] Bates R A critical analysis of evaluation practice: the Kirkpatrick model and the principle of beneficence. *Eval Program Plann*. 2004;27:341–347.

[21] Tamkin P Yarnall J Kerrin M Kirkpatrick and beyond: a review of training evaluation. *IES Report Number*. 2002;392.

[22] Jeffcott SA Evans SM Cameron PA, Improving measurement in clinical handover. *BMJ Qual Saf*. 2009;18:272–276. 10.1136/qshc.2007.024570 19651930

[23] Sarac C Flin R Mearns K, Hospital survey on patient safety culture: psychometric analysis on a Scottish sample. *BMJ Qual Saf*. 2011;20:842–848. 10.1136/bmjqs.2010.047720 21690247

